# Entropy Generation Rate Minimization for Methanol Synthesis via a CO_2_ Hydrogenation Reactor

**DOI:** 10.3390/e21020174

**Published:** 2019-02-13

**Authors:** Penglei Li, Lingen Chen, Shaojun Xia, Lei Zhang

**Affiliations:** 1 College of Power Engineering, Naval University of Engineering, Wuhan 430033, China; 2 Institute of Thermal Science and Power Engineering, Wuhan Institute of Technology, Wuhan 430205, China; 3 School of Mechanical & Electrical Engineering, Wuhan Institute of Technology, Wuhan 430205, China

**Keywords:** methanol synthesis via CO_2_ hydrogenation, plug flow reactor, entropy generation rate minimization, optimal control theory, finite time thermodynamics

## Abstract

The methanol synthesis via CO_2_ hydrogenation (MSCH) reaction is a useful CO_2_ utilization strategy, and this synthesis path has also been widely applied commercially for many years. In this work the performance of a MSCH reactor with the minimum entropy generation rate (EGR) as the objective function is optimized by using finite time thermodynamic and optimal control theory. The exterior wall temperature (EWR) is taken as the control variable, and the fixed methanol yield and conservation equations are taken as the constraints in the optimization problem. Compared with the reference reactor with a constant EWR, the total EGR of the optimal reactor decreases by 20.5%, and the EGR caused by the heat transfer decreases by 68.8%. In the optimal reactor, the total EGRs mainly distribute in the first 30% reactor length, and the EGRs caused by the chemical reaction accounts for more than 84% of the total EGRs. The selectivity of CH_3_OH can be enhanced by increasing the inlet molar flow rate of CO, and the CO_2_ conversion rate can be enhanced by removing H_2_O from the reaction system. The results obtained herein are in favor of optimal designs of practical tubular MSCH reactors.

## 1. Introduction

Over the past century, a huge amount of CO_2_ produced through burning fossil fuels has been released into the atmosphere, which has led to global warming. Nowadays, much effort is being put into carbon emission reduction. There are mainly three ways to reduce carbon emissions: (1) Utilizing clean energy sources, such as the solar, wind, nuclear, and tidal energy; (2) utilizing carbon sequestration and storage technology; (3) collecting and recycling CO_2_ through chemical reactions. The methanol synthesis via CO_2_ hydrogenation (MSCH) reaction is in fact an effective scheme for alleviating the greenhouse effect. What is more, methanol is a primary liquid petrochemical, and is widely used in the chemical and energy fields in applications such as hydrogen storage, dimethyl ether and hydrocarbon production, etc. [1,2,3,4].

However, the methanol synthesis via CO_2_ hydrogenation reaction still has some problems to be solved, e.g., high energy-consumption, low conversion rate and poor selectivity [5]. So far the studies for MSCH mainly include: (1) developing new catalysts and establishing the corresponding kinetic models [6,7,8]; (2) improving the MSCH reaction process by modeling and simulation [9,10,11,12]; (3) studying the thermodynamic performance of the MSCH reaction based on classical thermodynamic theory [5,13,14].

Thermodynamic equilibrium and reversibility are the main assumptions in classical thermodynamic analysis. The plug flow reactor studied herein involves three irreversible phenomena: the chemical reaction, viscosity flow, and heat transfer, which result in entropy generation [15]. Therefore, the MSCH reactor should been analyzed and optimized based on finite time thermodynamic (FTT) theory [16,17,18,19,20,21,22,23,24,25,26,27,28,29], which has been utilized to optimize irreversible processes and cycles considering the effect of the finite time or size. In the engineering field it is also known as entropy generation minimization [30,31,32,33,34].

Many scholars have generally preferred to make the yield of the target production as the optimization objective function in their performance studies on industrial reactors. In 1986, Månsson and Andresen [35] firstly utilized FTT theory to study the performance of a catalyzed ammonia synthesis reactor and obtained the optimal temperature profile of reaction mixtures corresponding to the objective function of the maximum ammonia yield. Jahanmiri and Eslamloueyan [36] studied the performance of a methanol synthesis reactor and obtained the optimal temperature profile of the reaction mixture corresponding to the objective function of the maximum methanol yield. Farsi and Jahanmiri [37,38,39,40] completed a series of works on the performance of the methanol synthesis membrane reactor for the sake of improving the methanol yield. Wang et al. [41] studied the performance of a sulphuric acid decomposition reactor with the maximum SO_2_ yield as the objective function and obtained the optimal exterior wall temperature (EWT) and pressure of reaction mixture (PRM) profiles.

According to Gouy-Stodola theory, the amount of lost work is defined as the product of ambient temperature and the entropy generation of process, so the irreversibility of the process will be reduced by minimizing the entropy generation of the irreversible process [31,32,33,42]. Kjelstrup et al. [43] utilized the MSCH kinetic model established by Bussche and Froment [44] to study the MSCH reactor and obtained the optimal temperature profile of reaction mixture corresponding to the objective function of minimum total entropy generation rate (EGR) caused by the MSCH reaction. Johannessen and Kjelstrup [45] studied the performance of SO_2_ oxidation reactor with the EGR minimization as the optimization objective and obtained the optimal EWT profile and reactor length. Nummedal et al. [46] studied the SMR reactor with the EGR minimization as the optimization objective and obtained the optimal EWT profile. Ao et al. [47] and Chen [48] et al. also studied the performances of SMR reactors with the heat transfer laws of linear phenomenological [47] and Dulong-Petit [48], respectively, and obtained the optimal EWT profiles. van der Ham et al. [42] optimized the performance of a sulfuric acid decomposition reactor and obtained two pathways to reduce the EGR in the reactor. Kingston and Razzitte [49,50] studied the EGR of two ideal reactors, the dimethyl ether synthesis reactor with the EGR minimization as the optimization objective and obtained the optimal inlet composition of the reactor. On the basis of these achievements, they also investigated the thermally coupled reactor. Chen et al. [51] established a tubular flow reactor model for the process of CO_2_ hydrogenation to light olefins and obtained the optimal design parameters with the minimum specific EGR as the objective function. Chen et al. [52] established a hollow fiber membrane contactor model for extracting CO_2_ from seawater and obtained the analytical formulae of the CO_2_ extraction rate and the EGR of the process. Zhang et al. [53] established a reverse water-gas shift reaction (RWGS) reactor model based on the experimental data and obtained the optimal configuration for the minimum total EGR of the reactor.

The previous works on the minimum EGR of the industrial reactors indicate that the total EGR of reactor can be reduced by adjusting the reactor length, improving the transport properties, and changing the operation condition. Changing the operation condition is considered as the only approach to reduce the total EGR in this paper, since the EGR caused by the viscous flow is insignificant, and the transport properties depend on the material and catalyst et al. [15]. Kjelstrup et al. [43] optimized the temperature profile corresponding to the minimum total EGR caused by the MSCH reaction. This paper will use FTT theory to optimize the performance of MSCH reactor with the total EGR minimization as the optimization objective and seek the optimal EWT profile and the optimal boundary conditions.

## 2. Reactor Model and System Description

### 2.1. Reactor Model

Two routes of methanol synthesis can be found in public literature, i.e., (1) CO_2_ shifts to CO through RWGS, and then CO convert into CH_3_OH; (2) CO_2_ convert into CH_3_OH through an intermediate HCOO [3]. Undergo the experimental research of many years, Skrzypel et al. [54] and Vanden Bussche and Froment [55] confirmed that CO_2_ is the main source of methanol synthesis, and CO and CO_2_ can mutual converts through the RWGS reaction [2,9]. Two reactions occurring in the reactor are as follows [9,36,44]:

(1) $${\mathrm{CO}}_{2}{+3H}_{2}\overset{}{⇌}{\mathrm{CH}}_{3}{\mathrm{OH}+H}_{2}O\;\;\;\;\Delta_{r}H_{1}<0$$

(2) $${\mathrm{CO}}_{2}{+H}_{2}\overset{}{⇌}\mathrm{CO}+H_{2}O\;\;\;\;\;\;\Delta_{r}H_{2}>0$$

where $\Delta_{r}H_{i}$ is the enthalpy of reaction i.

**Figure 1:**
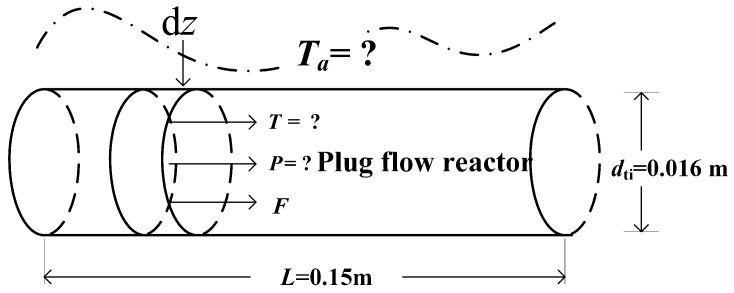
One-dimensional pseudo-homogeneous plug flow MSCH reactor model.

In the chemical industrial production, two reactions generally reacts in a suit of catalyst-filled tubes, which are placed inside a tubular heat exchanger. In order to simplify the model, this paper will study one such tube, and establish a one-dimensional pseudo-homogeneous plug flow reactor model as shown in Figure 1 [15,35,41,42,43,44,45,46,47,48,49,50,51,52,53].

According to the judgmental criterion of the assumption on the plug flow, one reactor can be taken as a plug flow reactor, in the case of $L/d_{\mathrm{ti}}$ (*L* and *d*_ti_ are, respectively, the reactor length and diameter) exceeds 150 [56]. Herein, $L/d_{\mathrm{ti}}$ is equal to 300, therefore, the MSCH reactor can be taken as a plug flow reactor. The mathematical model of diffusion listed in Appendix A, Appendix B, and Appendix C are consistent with recent treatments of reaction-diffusion descriptions by Niven [57] and England [58] and a recent temperature-dependent description by Ritchie [59]. The effect of the diffusion within the catalyst pellet on the chemical reaction rate can be ignored, when the effectiveness factor of chemical reaction is close to 1. In this paper, the diffusion within the catalyst pellet can be ignored, and the MSCH reactor can be taken as a pseudo-homogeneous reactor through the verification. The detailed discussion is shown in Appendix A and Appendix B. The MSCH reactor herein can be studied with one-dimensional model, since the size of MSCH reactor is not large, and radial gradient of temperature and mass can be ignored. In summary, the MSCH reactor model herein includes following assumptions: (1) the reaction mixture is not back-mixed in the axial, and mixes uniformly in the radial; (2) the radial temperature and concentration gradients are neglected.

In the MSCH reactor, both the reactants and products are taken as the ideal gas. The reactor geometry parameters, the ICI 51-2 Cu/ZnO/Al_2_O_3_ catalyst geometry parameters, the physical parameters of mixture gas and the inlet conditions derived from the reference reactor in References [44,45] are listed in Table 1. The overall heat transfer profile of the reference reactor along the dimensionless axial can be obtained based on the empirical formula proposed by Dixonge [60,61], and the calculation results indicate that the overall heat transfer coefficient is approximately equal to 60 $W/\left(K\cdot m^{2}\right)$.

### 2.2. Reaction Kinetic Model

The MSCH reaction processes can be classified as the high-pressure process, 25–30 MPa, the medium-pressure process, 10–25 MPa, and the low-pressure process, 5–10 MPa [36]. Among them, there are more researches on low-pressure process, since the low-pressure of methanol synthesis process is applied widely in chemical industries [1,3,62].

In this paper, the kinetic model established by Vanden Bussche and Froment [44] is selected and utilized, since this kinetic model is based on the ICI 51-2 Cu/ZnO/Al_2_O_3_ catalyst, which is applied widely in chemical industries, such as the LURGI type methanol synthesis reactor [9]. Another reason is that the kinetic model proposed by Reference [44] has been checked by experiments of lab-scale [44,55] and commercial-scale [9], what is more, the kinetic model has a wider application range. The temperature varies between 180 and 280 °C, the pressures varies between 15 and 51 bar in the experiment of Vanden Bussche and Froment [44]. The pressure from industrial data of LURGI type methanol synthesis reactor is 66.7 bar [9].

According to the kinetic model proposed by Reference [44], the reaction rates of the MSCH and RWGS reactions are:

(3) $$r_{1}=\kappa_{1}P_{{\mathrm{CO}}_{2}}P_{H_{2}}[1-(1/K_{1}^{*})(P_{H_{2}O}P_{{\mathrm{CH}}_{3}\mathrm{OH}}/P_{H_{2}}^{3}P_{{\mathrm{CO}}_{2}})]\beta^{3}$$

(4) $$r_{2}=\kappa_{2}P_{{\mathrm{CO}}_{2}}[1-K_{2}^{*}(P_{H_{2}O}P_{\mathrm{CO}}/P_{{\mathrm{CO}}_{2}}P_{H_{2}})]\beta$$

(5) $$\beta =1/[1+\kappa_{3}(P_{H_{2}O}/P_{H_{2}})+\kappa_{4}\sqrt{P_{H_{2}}}+\kappa_{5}P_{H_{2}O}]$$

(6) $$\log_{10}(K_{1}^{*})=\frac{3066}{T}-10.592$$

(7) $$\log_{10}(K_{2}^{*})=\frac{-2073}{T}+2.029$$

where $r_{i}$ is the reaction rate of reaction i, $P_{k}=(PF_{k})/F_{T}$ is the partial pressure of component k, P and T are the pressure and temperature of reaction mixture (TRM), respectively, $F_{k}$ and $F_{T}$ are the mole flow rate of component *k* and reaction mixture in the axial position z, respectively, $K_{1}^{*}$ and $K_{2}^{*}$ are thermodynamic equilibrium constants of Reactions (1) and (2) [63], respectively, and $\kappa_{j}$ is parameter group j related to the adsorption, equilibrium, and rate constants of elementary reactions. These parameter groups can be calculated as follows [44]:

(8) $$\kappa_{j}=A(j)\exp[B(j)/R_{g}T]$$

where $R_{g}=8.314\;J/\left(\mathrm{mole}\cdot K\right)$ is the universal gas constant, A(i) is the frequency factor, B(i) represents either E or −ΔH, and these factors are listed in Table 2 [44].

### 2.3. Conservation Equation

The heat transfer between the reaction mixture and the exterior wall heat reservoir obeys the Newton heat transfer law, i.e., q∝(ΔT), and the heat flux passed through the tube wall is

(9) $$J_{q}=U(T_{a}-T)$$

where U is the overall heat transfer coefficient, and $T_{a}$ is the EWT.

The energy conservation equation is:

(10) $$\frac{dT}{dz}=\frac{\pi d_{\mathrm{ti}}J_{q}-\rho_{c}A_{c}\left(1-\varepsilon_{c}\right)\sum_{i}r_{i}\Delta_{r}H_{T,i}}{\sum_{k}F_{k}C_{p,k}}$$

where $\varepsilon_{c}$ is the void fraction of the catalyst bed, $\rho_{c}$ is the catalyst density, $C_{p,k}$ is the mole heat capacity at constant pressure of the component k, and $\Delta_{r}H_{T,i}$ is the standard mole enthalpy of reaction i. These parameters can be obtained as follows [64]:

(11) $$C_{p,k}=A_{k}+B_{k}T+C_{k}T^{2}+D_{k}T^{3}+E_{k}T^{4}$$

(12) $$\Delta_{r}H_{T,i}=\sum_{k}\left(\upsilon_{k,i}\Delta_{f}H_{T,\;k}+I_{i}\right)$$

(13) $$\Delta_{f}H_{T,\;k}=A_{k}T+(\upsilon_{k,i}B_{k}T^{2})/2+(\upsilon_{k,i}C_{k}T^{3})/3+(\upsilon_{k,i}D_{k}T^{4})/4+(\upsilon_{k,i}E_{k}T^{5})/5$$

(14) $$I_{i}=\sum_{k}\upsilon_{k,i}\left(\Delta_{f}H_{298.15K,\;k}^{0}-\Delta_{f}H_{298.15K,\;k}\right)$$

where $\upsilon_{k,i}$ is the stoichiometric number of component k in reaction i, $I_{i}$ is the integration constant of reaction i that can be obtained based on the standard mole enthalpy $\Delta_{f}H_{298.15K,\;k}^{0}$ and the coefficients A, B, C, D, E, $\Delta_{f}H_{298.15K}^{0}$ and $M_{k}$ are listed in Table 3 [65].

The momentum equation utilized to describe the pressure drop along the reactor axial is generally described by the Ergun equation, in the case of $Re_{p}/(1-\varepsilon_{c})<500$ [66]. The Reynolds numbers $Re_{p}$ is:

(15) $$Re_{p}=Gd_{p}/\mu_{\mathrm{mix}}$$

where G is the superficial mass flow rate, $G=\sum_{k}\left(F_{k}M_{k}\right)/A_{c}$, $M_{k}$ is the mole mass of component k, $\mu_{\mathrm{mix}}$ is the viscosity of reaction mixture (see Appendix C), and $d_{p}$ is the catalyst particle diameter.

$Re_{p}/(1-\varepsilon_{c})$ is less than 0.005 in the case discussed in this paper, therefore, the momentum equation can be described by the Ergun equation as follows:

(16) $$\frac{dP}{\mathrm{dz}}=-\left[\frac{150\mu_{\mathrm{mix}}(1-\varepsilon_{c})}{d_{p}^{}}+1.75G\right]\frac{c_{g}}{d_{p}}\frac{\left(1-\varepsilon_{c}\right)}{\varepsilon^{3}}$$

where $c_{g}=F_{T}R_{g}T/(PA_{c})$ is the superficial velocity of mixture gas.

The mole balance equations are described by the yields of CH_3_OH and CO ($\xi_{1}$ and $\xi_{2}$), which are both defined with the inlet mole flow rate of CO_2_ [15]:

(17) $$\frac{d\xi_{1}}{\mathrm{dz}}=\rho_{c}A_{c}\left(1-\varepsilon_{c}\right)\frac{r_{1}}{F_{{\mathrm{CO}}_{2},\mathrm{in}}}$$

(18) $$\frac{d\xi_{2}}{\mathrm{dz}}=\rho_{c}A_{c}\left(1-\varepsilon_{c}\right)\frac{r_{2}}{F_{{\mathrm{CO}}_{2},\mathrm{in}}}$$

where subscript ‘in’ denotes the inlet state of variables, and the mole flow rate of component k and the total mole flow rate are defined as follows:

(19) $$F_{k}=F_{k,\mathrm{in}}+F_{{\mathrm{CO}}_{2},\mathrm{in}}\sum_{i}\upsilon_{i,k}\xi_{i}$$

(20) $$F_{T}=F_{T,\mathrm{in}}-2F_{{\mathrm{CO}}_{2},\mathrm{in}}\xi_{1}$$

### 2.4. Entropy Generation Rate of the MSCH Reactor

Non-equilibrium thermodynamics indicates that the irreversible process always generates EGR, which is described as the product sum of the conjugate fluxes and forces [67,68,69]. In a plug flow reactor, the EGR is mainly produced by the chemical reactions, heat transfer and viscous flow [15,67,68,69].

In term of the EGR due to chemical reactions, the driving forces are:

(21) $$-\frac{\Delta_{r}G_{1}}{T}=-R_{g}\ln\frac{P_{{\mathrm{CH}}_{3}\mathrm{OH}}P_{H_{2}O}^{}}{P_{{\mathrm{CO}}_{2}}P_{H_{2}}^{3}K_{1}^{*}}$$

(22) $$-\frac{\Delta_{r}G_{2}}{T}=-R_{g}\ln\frac{P_{\mathrm{CO}}P_{H_{2}O}}{P_{{\mathrm{CO}}_{2}}P_{H_{2}}K_{2}^{*}}$$

where $\Delta_{r}G_{i}$ is the Gibbs free energy of reaction i.

In term of the EGR due to heat transfer, the driving force is:

(23) $$\Delta (1/T)=\frac{1}{T}-\frac{1}{T_{a}}$$

In term of the EGR due to viscous flow, the driving force is:

(24) $$-\frac{dP}{T\mathrm{dz}}$$

The local EGR is:

(25) $$\sigma_{\mathrm{TOT}}=\pi d_{ti}J_{q}\left(\frac{1}{T}-\frac{1}{T_{a}}\right)+\rho_{c}A_{c}\left(1-\varepsilon \right)\sum_{i}r_{i}\left(\frac{-\Delta_{r}G_{i}}{T}\right)-A_{c}c_{g}\frac{1}{T}\frac{dP}{\mathrm{dz}}$$

where the three terms on right side are the local EGR due to heat transfer, $\sigma_{H}$, MSCH reaction, $\sigma_{\mathrm{MS}}$, RWGS reaction, $\sigma_{\mathrm{RW}}$, and viscous flow, $\sigma_{F}$, respectively, and the total EGR, ${\left(dS/dt\right)}_{\mathrm{TOT}}$ obtained by the integral of the local EGR along the reactor axial is as follows:

(26) $${\left(\frac{dS}{dt}\right)}_{\mathrm{TOT}}=\int_{0}^{L}\sigma_{\mathrm{TOT}}\left(z\right)\mathrm{dz}=\sum_{H}+\sum_{\mathrm{MS}}+\sum_{\mathrm{RW}}+\sum_{F}$$

where $\sum_{H}$, $\sum_{\mathrm{MS}}$, $\sum_{\mathrm{RW}}$ and $\sum_{F}$ are, respectively, the total EGR due to heat transfer, MSCH reaction, RWGS reaction and viscous flow.

The optimization objective is:

(27) $$\mathrm{Min}\;{\left(\frac{dS}{dt}\right)}_{\mathrm{TOT}}=\mathrm{Min}\left[\int_{0}^{L}\sigma_{\mathrm{TOT}}\left(z\right)dz\right]$$

Equation (27) takes into account almost all phenomena generating EGR in the MSCH reactor, while Reference [43] only considered the EGR from the MSCH reaction. Therefore, the study for the optimal reactor herein is more comprehensive, and the optimal results can be favorable for the optimal design for the practical MSCH reactor.

## 3. Mathematical Description of the Optimization Problem

The optimization problem herein is to minimize the total EGR subjected to some constraints. The fixed methanol yield ($\xi_{1}=1.3139$, $\Delta F_{{\mathrm{CH}}_{3}\mathrm{OH}}=1.32\times 10^{-4}$) and the conservation equations are taken as the constraints. The control variable, i.e., the EWT can be controlled completely. The inlet PRM and the inlet mole flow rate of components in the optimal reactor are same with those in the reference reactor [43]. At the outlet, all variables except for the methanol yield are allowed to change freely. The geometry sizes and the catalyst properties are same with those of the reference reactor in Reference [43]. Optimal control theory is utilized to solve this optimization problem and to find the optimal EWT profile.

### 3.1. Application of Optimal Control Theory

In the early 1950s, optimal control theory had been applied in the study of the minimum time control problem. However, the original optimal control theory cannot solve an optimization problem that the admissible control belongs to a closed set. In order to solve this problem, Pontryagin created the minimum principle on 1958 [70].

The purpose of this paper is to minimize the total EGR of the MSCH reactor and find the optimal EWT profile by using optimal control theory. Therefore, the EWT is taken as the control variable, the total EGR is taken as the performance objective, and the TRM (T), PRM (P), methanol yield ($\xi_{1}$), and carbon monoxide yield ($\xi_{2}$) are taken as the state variables controlled by the conservation equations. The inlet and outlet values of state variables and the multiplier functions corresponding to the state variables are taken as the boundary condition [15].

The Hamiltonian can be established as follow [15,42,70]:

(28) $$H=\sigma_{\mathrm{TOT}}+\lambda_{T}\frac{dT}{dz}+\lambda_{P}\frac{dP}{dz}+\lambda_{\xi_{1}}\frac{d\xi_{1}}{dz}+\lambda_{\xi_{2}}\frac{d\xi_{2}}{dz}$$

where $\lambda_{T}$, $\lambda_{P}$, $\lambda_{\xi_{1}}$, and $\lambda_{\xi_{2}}$ are the multiplier functions of state variables.

According to the minimum principle of Pontryagin [70], the necessary conditions for the EGR minimization, i.e., canonical Equations, are as follows:

(29) $$\partial T/\partial z=\partial H/\partial \lambda_{T}$$

(30) $$\partial P/\partial z=\partial H/\partial \lambda_{P}$$

(31) $$\partial \xi_{1}/\partial z=\partial H/\partial \xi_{1}$$

(32) $$\partial \xi_{2}/\partial z=\partial H/\partial \xi_{2}$$

(33) $$\partial \lambda_{T}/\partial z=-\partial H/\partial T$$

(34) $$\partial \lambda_{P}/\partial z=-\partial H/\partial P$$

(35) $$\partial \lambda_{\xi_{1}}/\partial z=-\partial H/\partial \xi_{1}$$

(36) $$\partial \lambda_{\xi_{2}}/\partial z=-\partial H/\partial \xi_{2}$$

where Equations (29)–(32) are state equations, and Equations (33)–(36) are adjoint Equations.

Hamiltonian also needs to satisfy the extremum condition, i.e., $\partial H/\partial T_{a}=0$. The relation between the EWT and the TRM can be derived via this extremum condition [15]:

(37) $$T_{a}=T{\left(1+\frac{\lambda_{T}T}{\sum_{k}F_{k}C_{p,k}}\right)}^{-1/2}$$

The boundary conditions of optimal control theory are obtained based on the transversal condition, i.e., $x_{n}=x_{n,\mathrm{specification}}\;\mathrm{or}\;\lambda_{n}=0$. The boundary conditions are listed in Table 4. The property of Hamilton [70] is often used to check the veracity of the optimal results. Hamiltonian herein keeps on a constant along the optimal trajectory, since it does not depend on explicitly the axial reactor length, i.e., Hamiltonian is autonomous.

### 3.2. Numerical Calculations of Optimization Problem

Solving the optimization problem must has a reasonable initial value. The calculation results of the reference reactor with constant heat reservoir $T_{a}=523\;K$ [43] solved by the numerical discretization are taken as the initial values of the optimal reactor. Optimization problem herein involves eight differential equations, i.e., (Equations (29)–(36)), 1 algebraic equation, i.e., Equation (37), and eight boundary conditions listed in Table 4. Therefore, the optimal control comes down to solve a two-point boundary value problem involving the differential equation. Optimal solution can be obtained by the ‘bvp4c’ solver in Matlab. The solution accuracy is mainly influenced by the initial multiplier functions, i.e., $\lambda_{\xi_{1}}$, $\lambda_{\xi_{2}}$, and the number of grid points. In order to improve the solution accuracy, this paper utilizes 5000 grid points. The calculation results show that the solution error does not exceed $6.0\times 10^{-8}$.

## 4. Numerical Results and Discussions

The reference reactor with the constant heat reservoir $T_{a}\;=\;523\;K$ and the optimal reactor with minimum EGR due to the MSCH reaction [43], i.e., the optimal reactor in [43] are both utilized to compare with the optimal reactor herein, i.e., the optimal reactor. In the three reactors, the geometry sizes of reactor, the catalyst properties, and the inlet components are the same with those of the reference reactor in Reference [43].

Table 5 lists the total EGRs of the three reactors. Compared with the results of the reference reactor in [43], the total EGR of the optimal reactor decreases by 20.5%, the total EGR due to the heat transfer decreases by 68.8%, the total EGR due to two chemical reactions decreases by 3.3%, and the total EGR due to the viscous flow is almost unchanged. In the reference and optimal reactors, the total EGRs due to the MSCH reaction account for 69% and 84% of the total EGR, respectively. In the optimal reactor of [43], the total EGR due to the MSCH reaction only accounts for 7.1% of the total EGR, however, the total EGR increases by 123.9% compared with that of the reference reactor.

**Figure 2:**
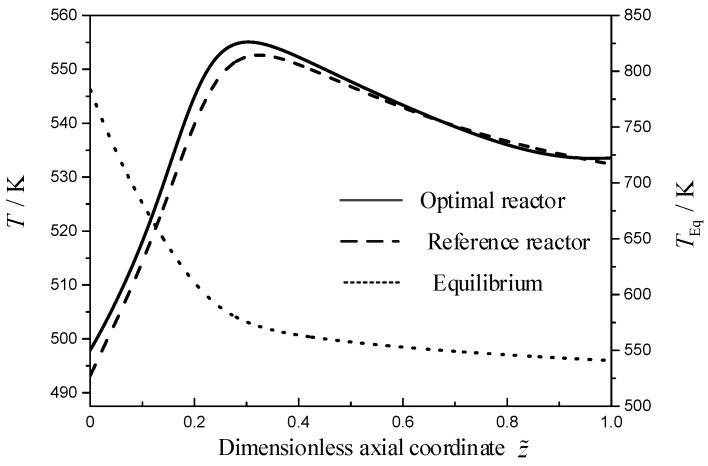
The TRM profiles for the two reactors, $T\left(\tilde{z}\right)$, and the equilibrium temperature of the MSCH reaction, $T_{\mathrm{eq}}\left(\tilde{z}\right)$.

**Figure 3:**
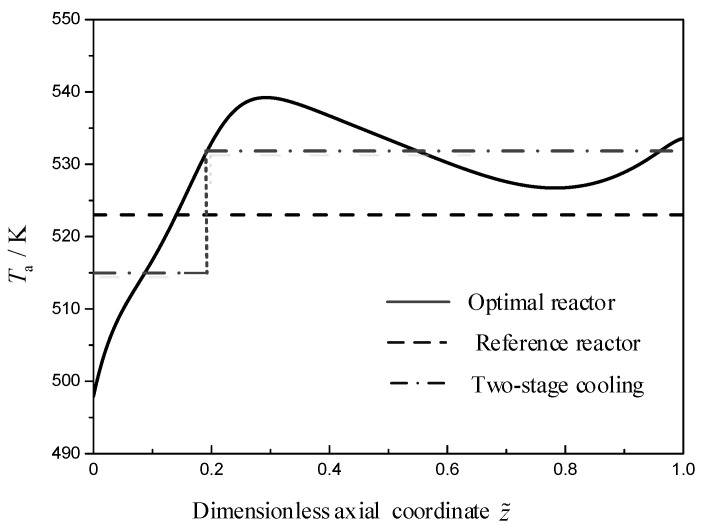
The EWT profiles for the reference, optimal, and two-stage cooling reactor, $T_{a}\left(\tilde{z}\right)$.

Figure 2 describes the TRM profiles of the reference and optimal reactors, and the equilibrium temperature profile of the MSCH reaction in reference reactor. As shown in Figure 2, the TRM in the reference reactor (dashed line) increases from 493.2 K at the reactor inlet up to a hot spot of 552.6 K, and then it decreases toward a value of 532.4 K. The TRM in the optimal reactor (solid line) increases from 497.9 K at the reactor inlet up to a hot spot of 555.1 K, next it decreases toward a value of 533.4 K, and then it increases toward a value of 533.5 K at the reactor outlet. Except for the range near the reactor outlet, the TRM profiles in the two reactors are almost the same. The above results can be explained according to the theory of the chemical reaction equilibrium, i.e., Le Chatelier’s principle. As shown in Figure 2, the equilibrium temperature of the MSCH reaction (the calculation results based on Equation (22)) decreases steeply from 783.6 K at the reactor inlet up to a turning point of 588 K at the dimensionless axial position $\tilde{z}=0.25$, and then levels off to a more flat decrease until a value of 540.9 K. Near to the inlet of the two reactors, the difference between the TRMs and equilibrium temperature is so large that the chemical driving force is large and the reaction rate is also large. Therefore, a large amount of heat is released by the MSCH reaction, which results into the steep increase of the TRM. With the decrease of the difference between the TRM and equilibrium temperature, the EWTs (as shown in Figure 3) become the dominating contribution to the TRMs. Therefore, the TRMs show a flat decrease under the cooling effect of the EWTs.

Figure 3 describes the EWT profiles of the reference and optimal reactors along the reactor axial. It is noteworthy that the optimal EWT profile (solid line) shown in Figure 3 is a significant result herein. In industrial, this ideal EWT profile can be reached approximately through assigning some heat exchanger providing different constant cooling temperature, e.g., two-stage cooling strategy (dash dot line) in this case can reduce the total EGR 10.78% with respect to the reference reactor. As shown in Figure 3, the EWT in reference reactor (dash line) shows a constant profile along the reactor axial. The EWT in the optimal reactor increases from 497.9 K up to a local maximum of 539.2 K at the dimensionless axial position $\tilde{z}=0.29$, next it decreases to a local minimum of 526.7 K at the dimensionless axial position $\tilde{z}=0.78$, and then it increases to a maximum of 533.6 K at the reactor outlet.

**Figure 4:**
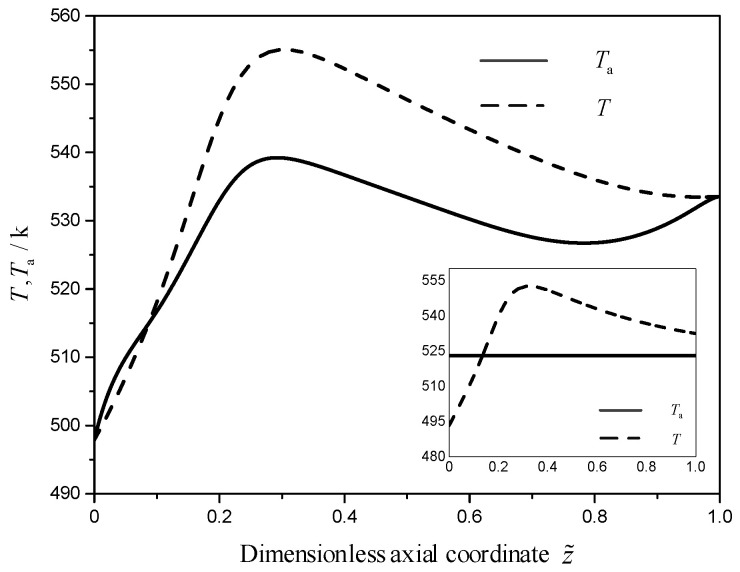
The EWT and TRM profiles for the reference (small frame) and. optimal reactors (large frame).

Figure 4 describes that the optimal EWT (solid line) and TRM (dashed line) profiles in the optimal (large frame) and reference reactors (small frame). As shown in large frame of Figure 4, the EWT is almost all less than the TRM. According to the Le Chatelier’s principle, decreasing the TRM is favor of increasing the carbon dioxide conversion and methanol production rates for the endothermic reaction. Therefore, the EWT should be lower than the TRM. It is noteworthy that the EWTs are equal to the TRMs at the inlet and outlet in the optimal reactor. The reason for this phenomenon is that the EWT is equal to the TRM, in the case of the TRM changes freely at the boundary according to optimal control theory.

**Figure 5:**
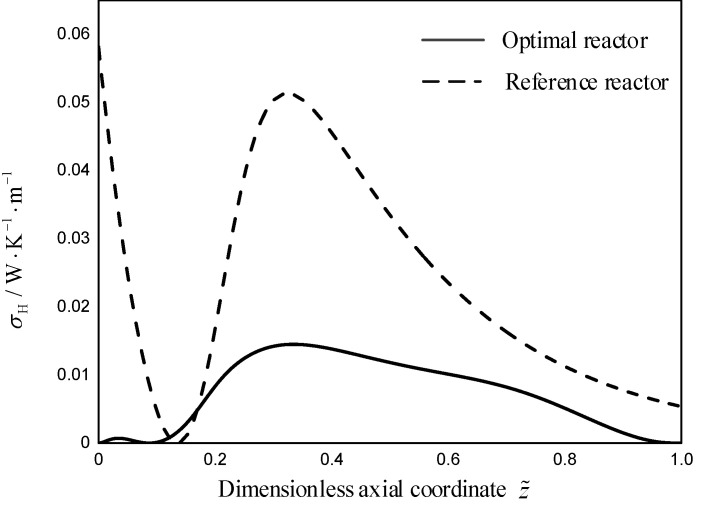
The local EGR due to heat transfer for the two reactors, $\sigma_{H}\left(\tilde{z}\right)$.

**Figure 6:**
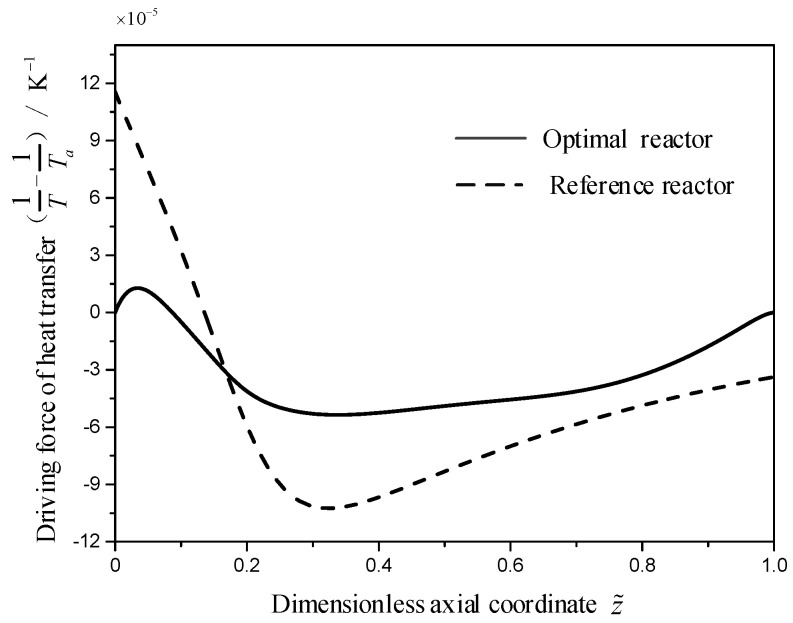
The thermodynamic driving force profiles, Δ(1/T) for the two reactors.

As shown in Figure 4, the temperature difference in the optimal reactor between the TRM and EWT is almost all less than that in the reference reactor. Therefore, the local EGR due to the heat transfer in the optimal reactor is less than that in the reference reactor (as shown in Figure 5). As shown in Figure 5 and Figure 6, compared with the reference reactor, the local EGR and driving force due to the heat transfer in the optimal reactor distribute more smooth which is in accordance with the equipartition principle of entropy generation and driving force [15,71,72,73,74].

**Figure 7:**
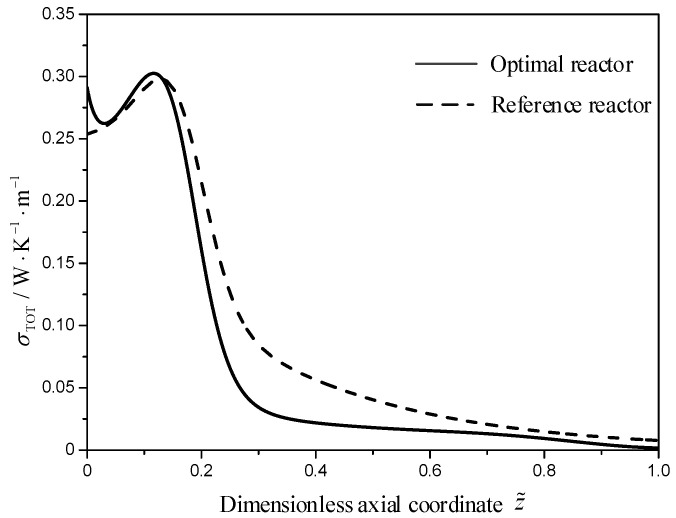
The local EGR profiles for the two reactors, $\sigma \left(\tilde{z}\right)$.

Figure 7 describes the local EGR profiles of the two reactors. As shown in Figure 7, more than 75% of the total EGR distributes in the first 30% length of the two reactors. The local EGR of the optimal reactor (solid line) decreases from 0.2911 W/(K·m) up to a local minimum of 0.2623 W/(K·m) at the dimensionless axial position $\tilde{z}=0.029$, next it increases toward a value of 0.3025 W/(K·m) at the dimensionless axial position $\tilde{z}=0.115$, next it decreases steeply toward a value of 0.028 W/(K·m) at the dimensionless axial position $\tilde{z}=0.33$, and then it levels off to a more flat decrease until a value of 0.002 W/(K·m) at the reactor outlet. Compared with the local EGR of the reference reactor (dash line), the local EGR of the optimal reactor distribute more even, especially after $\tilde{z}=0.33$. At the first 30% length of the two reactors, the dominating contribution for the local EGR comes from the MSCH reaction, after 30% length, the dominating contribution for the local EGR comes from the heat transfer.

**Figure 8:**
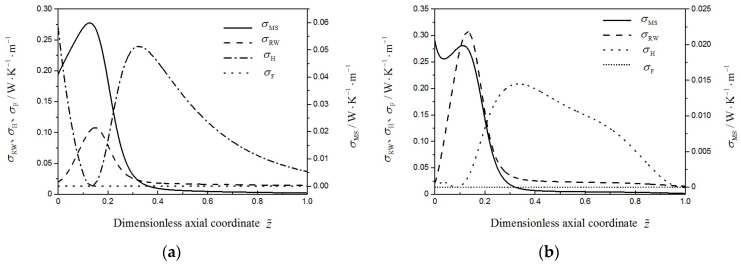
The local EGR due to the MSCH reaction, $\sigma_{\mathrm{MS}}\left(\tilde{z}\right)$, RWGS reaction, $\sigma_{\mathrm{RW}}\left(\tilde{z}\right)$, heat transfer, $\sigma_{H}\left(\tilde{z}\right)$, and viscous flow, $\sigma_{F}\left(\tilde{z}\right)$, for two reactors. (**a**) Reference reactor; (**b**) Optimal reactor.

Figure 8 describes the contributions of the heat transfer (dotted line), viscous flow (shot dot line), MSCH reaction (solid line) and RWGS reaction (dashed line) on the local EGR in the two reactors. As shown in Figure 8, the contribution of the MSCH reaction on local EGRs are maximal in the two reactors. After the dimensionless axial position $\tilde{z}=0.35$, the dominating contribution for the local EGR comes from the heat transfer. The local EGR caused by the viscous flow is minimal, since the size of the lab-scale reactor herein is so small that the pressure drop is negligible. However, the local EGR due to viscous flow cannot be ignored in practical methanol synthesis reactor. It is noteworthy that compared with other profiles, the local EGR profiles due to the heat transfer change most dramatically. The reason of this phenomenon is that under the constraints of fixed methanol yield and fixed reactor, the EGR due to heat transfer can be minimized in a larger optimization potential.

**Figure 9:**
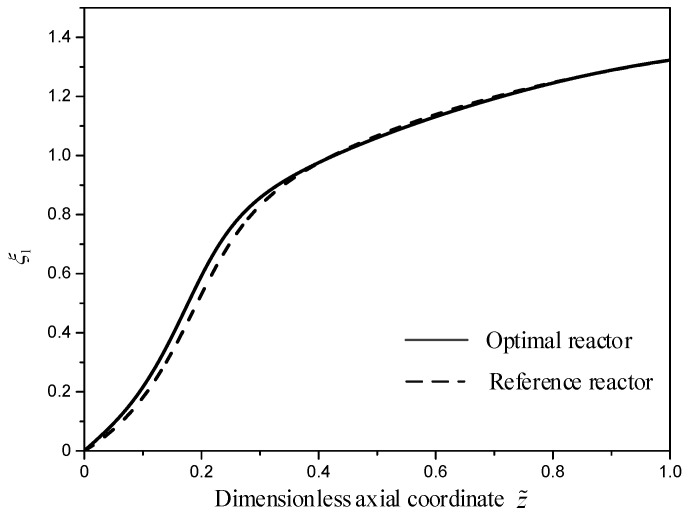
The methanol yield profiles, $\xi_{1}\left(\tilde{z}\right)$ for the two reactors.

Figure 9 describes the methanol yields of the two reactors. As shown in Figure 9, the profiles of the methanol yields of the two reactors are similar, since the outlet methanol yields of the two reactors are fixed. The inlet methanol yield is slightly larger than that of the reference reactor, since the initial MSCH reaction rate in the optimal reactor is slightly larger than that in the reference reactor.

**Figure 10:**
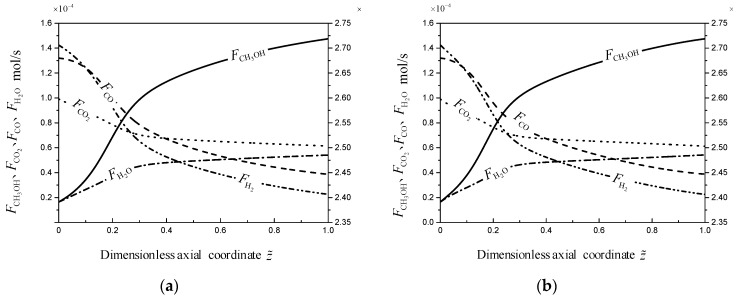
The mole flow rate profiles of component k For the two reactors, $F_{k}\left(\tilde{z}\right)$. (**a**) Reference reactor; (**b**) Optimal reactor.

Figure 10 describes mole flow rates of components in the two reactors. The mole flow rate of N_2_ is not given, since N_2_ doesn’t participate in the MSCH and RWGS reactions. As shown in Figure 10, the mole flow rate of CO decreases over the overall reactor, since the concentration of CO in reaction mixture exceeds the equilibrium concentration of CO under the TRM, which results in that the reaction direction of RWGS is changed. Undoubtedly, the reverse reaction of the RWGS reaction is beneficial for improving the reaction selectivity. The mole flow rate of H_2_O increases over the overall reactor. During the reaction process, with the increase of H_2_O, the MSCH reaction tends to reach the chemical equilibrium, which goes against the increase of methanol yield.

## 5. Conclusions

This paper studies the performance of MSCH reactor and obtains the optimal configuration of the EWT using FTT theory. The minimum EGR caused by heat transfer, viscous flow and chemical reaction is taken as the optimization objective, the fixed methanol yield, the fixed inlet pressure, and the fixed inlet components are taken as the constraints, and the completely controllable EWT is taken as the control variable. The mathematical model of the optimization problem is established using optimal control theory. The optimal results indicate that the total EGR decreases by 20.5% and the EGR due to heat transfer decreases by 68.8% compared with that of the reference reactor with a constant EWT profile. The local EGRs of the two reactors mainly distribute in the first 30% reactor length, and the EGRs due to the MSCH reaction account for more than 75%. The local EGR and driving force due to heat transfer in the optimal reactor distribute more evenly, which accords with the principle of equipartition of the entropy generation and driving force. With the increase of CO at reactor inlet, the CH_3_OH selectivity will increase. The CO_2_ conservation rate can be enhanced by removing H_2_O produced in the reaction process. The results obtained herein are in favor of the optimal designs of practical tubular reactors. In order to increase the exergy efficiency of the methanol system, the minimum EGR of the overall system involving the chemical reactor, heat exchanger, compressor, et. al. will be taken as the optimization objective in the future work.

## Figures and Tables

**Table 1:** Design parameters of the reference reactor [43].

Parameter	Sign	Value
Inlet temperature of reaction mixture	$T_{\mathrm{in}}$	493.2 K
Overall heat transfer coefficient	U	$60\;W/(K\cdot m^{2})$
Inlet total pressure	$P_{\mathrm{in}}$	$8.5\times 10^{6}\;\mathrm{Pa}$
Catalyst density	$\rho_{c}$	$1775\;{\mathrm{kg}/m}^{3}$
Catalyst void fraction	$\varepsilon_{p}$	0.5
Catalyst pellet diameter	$d_{p}$	$5\times 10^{-4}\;m$
Total catalyst weight	$W_{c,\mathrm{Tot}}$	0.0267 kg
Reactor length	L	0.15 m
Reactor diameter	$d_{\mathrm{ti}}$	0.016 m
Inlet total mole flow rate	$F_{T,\mathrm{in}}$	0.0033 mol/s
Inlet mole fraction of CO_2_	$x_{{\mathrm{CO}}_{2},\;\mathrm{in}}$	0.03
Inlet mole fraction of H_2_	$x_{H_{2},\mathrm{in}}$	0.82
Inlet mole fraction of CO	$x_{\mathrm{CO},\mathrm{in}}$	0.04
Inlet mole fraction of H_2_O	$x_{H_{2}O,\mathrm{in}}$	0.005
Inlet mole fraction of CH_3_OH	$x_{{\mathrm{CH}}_{3}\mathrm{OH},\;\mathrm{in}}$	0.005
Inlet mole fraction of N_2_	$x_{N_{2},\;\mathrm{in}}$	0.10

**Table 2:** Kinetic model parameters [44] *.

$\kappa_{j}$	A(j),B(j)	Value	$\kappa_{j}$	A(j),B(j)	Value
$k_{1}=k_{5a}^{\prime }K_{2}^{\prime }K_{3}K_{4}K_{H_{2}}$	*A*	1.07	$k_{2}=k_{1}^{\prime }$	*A*	$1.22\times 10^{10}$
	*B*	36,696		*B*	−94,765
$k_{3}=K_{H_{2}O}/K_{8}K_{9}K_{H_{2}}$	*A*	3453.38	$k_{4}=\sqrt{K_{H_{2}}}$	*A*	0.499
	*B*	-		*B*	17,197
$k_{5}=K_{H_{2}O}$	*A*	$6.62\times 10^{-11}$	-	-	-
	*B*	124,119	-	-	-

* $k_{5a}^{\prime }$ and $k_{1}^{\prime }$ are, respectively, the rate constants of the rate-determining steps for the MSCH and RWGS reactions, $K_{H_{2}}$ and $K_{H_{2}O}$ are, respectively, the adsorption constants of H_2_ and H_2_O, $K_{m}$s (m = 2, 3, 4, 8, 9) are the equilibrium constants of all elementary reactions except for some ignored.

**Table 3:** Thermodynamic parameters [66].

	$\begin{array}{l} k=1\\ \left({\mathrm{CO}}_{2}\right) \end{array}$	$\begin{array}{l} k=2\\ \left(H_{2}\right) \end{array}$	$\begin{array}{l} k=3\\ (\mathrm{CO}) \end{array}$	$\begin{array}{l} k=4\\ \left({\mathrm{CH}}_{3}\mathrm{OH}\right) \end{array}$	$\begin{array}{l} k=5\\ {(H}_{2}O) \end{array}$	$\begin{array}{l} k=6\\ \left(N_{2}\right) \end{array}$
$A_{k}$	27.4370	25.3990	29.5560	40.0460	33.9330	29.3420
$B_{k}(\times 10^{-3})$	42.3150	20.1780	−6.5807	−3.8287	−8.4186	−3.5395
$C_{k}(\times 10^{-5})$	−1.9555	−3.8549	2.0130	24.5290	2.9906	1.0076
$D_{k}(\times 10^{-8})$	3.9968	31.8800	−12.2270	−216.7900	−17.8250	−4.3116
$E_{k}(\times 10^{-11})$	−2.9872	−87.5850	22.6170	599.0900	36.9340	2.5935
$\Delta_{f}H_{298.15K}$ (kJ/mol)	−393.50	0	−110.50	−201.17	−241.80	191.6
$M_{k}\;(g/\mathrm{mol})$	44.01	2.016	28.01	32.042	18.015	28.013

**Table 4:** Boundary conditions of optimal control problems *.

	T	P	$\xi_{1}$	$\xi_{2}$
x(0)	-	85 bar	0	0
λ(0)	0	-	-	-
x(z)	-	-	1.323	-
λ(z)	0	0	-	0

* x(0) and x(z) are, respectively, the inlet and outlet values of state variables, λ(0) and λ(z) are, respectively, the multiplier functions of the corresponding state variables at the inlet and outlet.

**Table 5:** EGR of the three reactors *.

dS/dt (W/K)	$T_{a}=\mathrm{const}$	$dS_{\mathrm{chem}}^{1}/dt=\min$ [45]	$dS_{T}/dt=\min$
Reaction 1	$1.66\times 10^{-3}$	$3.80\times 10^{-4}$	$1.60\times 10^{-3}$
Reaction 2	$1.05\times 10^{-4}$	$3.71\times 10^{-5}$	$9.94\times 10^{-5}$
Heat transfer	$6.28\times 10^{-4}$	$4.94\times 10^{-3}$	$1.96\times 10^{-4}$
Viscous flow	$1.89\times 10^{-7}$	-	$1.90\times 10^{-7}$
Total EGR	$2.39\times 10^{-3}$	$5.35\times 10^{-3}$	$1.90\times 10^{-3}$

* The symbols $T_{a}=\mathrm{const}$, $dS_{\mathrm{chem}}^{1}/dt=\min$, and $dS_{T}/dt=\min$ represent the reference reactor and optimal reactor in Reference [45], and the optimal reactor herein, respectively.

**Figure A1:**
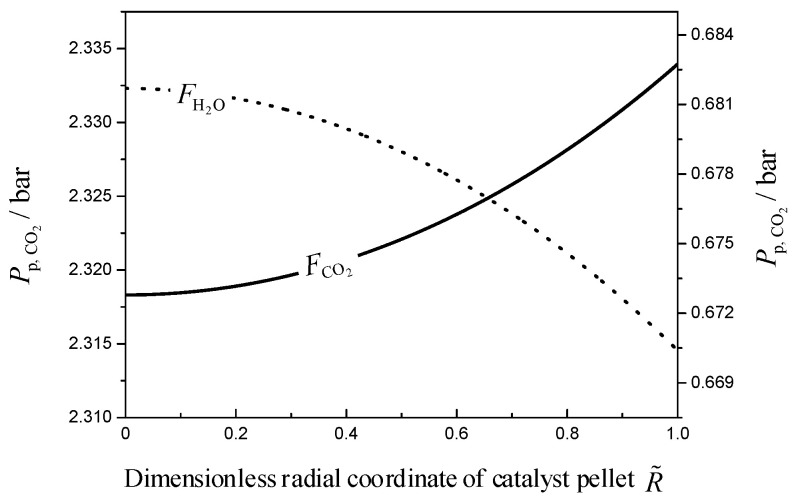
The partial pressure profiles of CO_2_, $P_{{p,\;\mathrm{co}}_{2}}\left(\tilde{R}\right)$, and H_2_O, $P_{{p,\;H}_{2}O}\left(\tilde{R}\right)$ inside the catalyst pellet.

**Figure A2:**
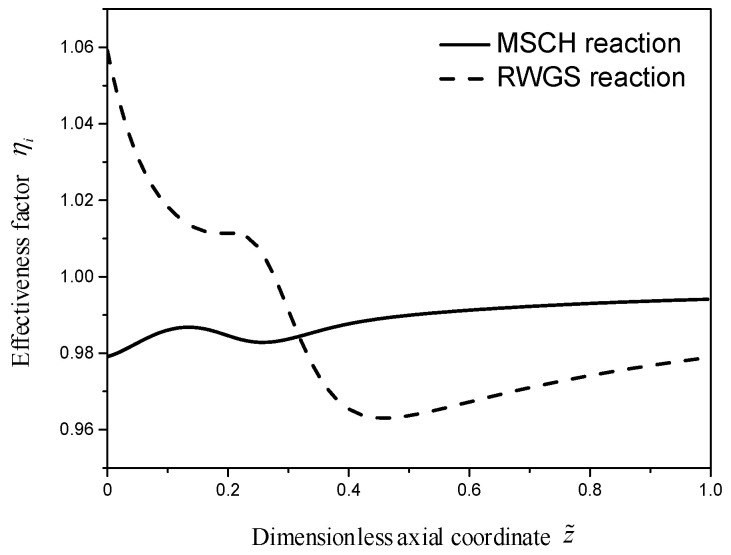
The effectiveness factor profiles for the MSCH and RWGS reaction, $\eta_{i}\left(\tilde{z}\right)$.

## Appendix A

The effectiveness factors $\eta_{1}$ and $\eta_{2}$ are taken as the value of 1. The reason of this phenomenon is that the complex MSCH reactor model involving the catalyst model, which cannot be optimized based on the optimal control. Therefore, the MSCH reactor is assumed to be a pseudo-homogeneous reactor. The reasonableness of $\eta_{1}=1$ and $\eta_{2}=1$ herein will be verified based on the MSCH reference reactor considering the catalyst pellet model.

The effectiveness factor $\eta_{i}$ of reaction i is defined as follow [75]

(A1) $$\eta_{i}=\frac{\int_{0}^{R_{p}}\left(R_{p}{}^{2}r_{p,\;i}\right)dR}{R_{p}{}^{3}r_{i}}$$

where $\eta_{i}$ is the effectiveness factor of reaction i, $R_{p}$ is the radius of the catalyst pellet, and $r_{i}$ and $r_{p,\;i}$ are the reaction rates in the catalyst bed and pellet, respectively. The $r_{i}$ and $r_{p,\;i}$ depend on the partial pressure in the catalyst bed $P_{k}$ and pellet $P_{p,\;k}$, respectively. The partial pressure profiles inside the catalyst pellet can be obtained based on the component balances equations as follows:

(A2) $$\frac{D_{m,k}^{e}}{R_{g}T_{}}\left(\frac{\partial^{2}P_{p,\;k}}{\partial R^{2}}+\frac{2}{R}\frac{\partial P_{p,\;k}}{\partial R}\right)+\rho_{c}\sum_{i=1}^{2}\upsilon_{k,i}r_{p,i\;}=0\;\;\;\;\;\;k=1,\ldots ,5$$

where $D_{m,k}^{e}$ is the effective diffusivities.

The boundary conditions at the pellet surface and at the center of the catalyst are as follows:

(A3) $$P_{p,k}=P_{k}\;\;\;\;\;\;\;\;\;\;\;\;R=R_{p}$$

(A4) $$\frac{\partial P_{p,k}}{\partial R}=0\;\;\;\;\;\;\;\;\;\;\;\;R=0$$

## Appendix B

Figure A1 describes the partial pressures of CO_2_ and H_2_O along the dimensionless radial coordinate of catalyst pellet at the dimensionless axial position $\tilde{z}=0.1$. As shown in Figure A1, the partial pressure of CO_2_ inside the catalyst pellet is less than that in the catalyst bed, i.e., $P_{p,{\;\mathrm{CO}}_{2}}\leq P_{{\mathrm{CO}}_{2}}$, when $\tilde{R}\leq 1$, while the partial pressure of H_2_O inside the catalyst pellet is more than that in the catalyst bed, i.e., $P_{p,{\;H}_{2}O}\geq P_{H_{2}O}$, when $\tilde{R}\leq 1$. The reason of this phenomenon is that the main reaction, i.e., MSCH reaction consumes CO_2_ and simultaneously produces H_2_O, and the diffusion rate of H_2_O is less than the formation rate of H_2_O inside the catalyst pellet.

Figure A2 describes the effectiveness factors in the reference reactor considering the catalyst model. As shown in Figure A2, the effectiveness factors of two reactions are close to the constant of 1, especially the effectiveness factor of the MSCH reaction (solid line). Table A1 lists the effect of the effectiveness factors on the key parameters of the reference reactor. As shown in Table A1, the effectiveness factors $\eta_{1}(z)$ and $\eta_{2}(z)$ in the reference reactor can be taken as 1. Therefore, the assumption of pseudo-homogeneous reactor is reasonable. It is noteworthy that the effectiveness factor of the RWGS reaction (dash line) is more than 1 between $\tilde{z}=0$ and $\tilde{z}=0.27$. The reason of this phenomenon is that, the reverse reaction rate of RWGS inside the catalyst pellet is more quickly than that in the catalyst bed, since the partial pressure of H_2_O inside the catalyst pellet is more than that in the catalyst bed.

**Table A1:** Evaluate the influence of actual effectiveness factors of reactions on the key parameters *.

Key Parameter	$\eta_{1}=\eta_{2}=1$	$\eta_{1}(z),\eta_{2}(z)$	Δ%
Methanol yield $\xi_{1}$	1.3139	1.318	0.3
Outlet TRM T/K	533	532	0.19
${\left(dS/dt\right)}_{\mathrm{TOT}}$ W/(K·m)	2.44$\times 10^{-3}$	2.42$\times 10^{-3}$	0.82

* where the symbol, $\eta_{1}=\eta_{2}=1$ and $\eta_{1}(z),\eta_{2}(z)$ represent the reference reactor model herein and the reference reactor model considering the catalyst pellet model, respectively.

## Appendix C

The viscosities of gases can be obtained from [65]:

(A5) $$\mu_{{\mathrm{CO}}_{2}}=11.811+4.9838\times 10^{-1}\cdot T-1.0851\times 10^{-4}\cdot T^{2}$$

(A6) $$\mu_{H_{2}}=27.758+2.1200\times 10^{-1}\cdot T-3.2800\times 10^{-5}\cdot T^{2}$$

(A7) $$\mu_{\mathrm{CO}}=23.811+5.3944\times 10^{-1}\cdot T-1.5411\times 10^{-4}\cdot T^{2}$$

(A8) $$\mu_{{\mathrm{CH}}_{3}\mathrm{OH}}=-14.236+3.8935\times 10^{-1}\cdot T-6.2762\times 10^{-5}\cdot T^{2}$$

(A9) $$\mu_{H_{2}O}=-36.826+4.2900\times 10^{-1}\cdot T-1.6200\times 10^{-5}\cdot T^{2}$$

(A10) $$\mu_{N_{2}}=42.606+4.7500\times 10^{-1}\cdot T-9.8800\times 10^{-5}\cdot T^{2}$$

The viscosity of mixture gas is derived from [76]:

(A11) $$\mu_{\mathrm{mix}}=\sum_{k=1}^{n}\frac{\mu_{k}}{\sum_{j=1}^{n}\phi_{k,j}\left(y_{j}/y_{k}\right)}$$

(A12) $$\phi_{k,j}=\frac{{\left[1+{\left(\mu_{k}/\mu_{j}\right)}^{1/2}{\left(M_{j}/M_{k}\right)}^{1/4}\right]}^{2}}{{\left[8\left(1+M_{k}/M_{j}\right)\right]}^{1/2}}$$

The thermal conductivities of gases can be obtained from [65]:

(A13) $$\lambda_{{\mathrm{co}}_{2}}=-0.012+1.0208\times 10^{-4}\cdot T-2.2403\times 10^{-8}\cdot T^{2}$$

(A14) $$\lambda_{H_{2}}=0.03951+4.5918\times 10^{-4}\cdot T-6.4933\times 10^{-8}\cdot T^{2}$$

(A15) $$\lambda_{\mathrm{CO}}=0.00158+8.2511\times 10^{-5}\cdot T-1.9081\times 10^{-8}\cdot T^{2}$$

(A16) $$\lambda_{{\mathrm{CH}}_{3}\mathrm{OH}}=0.00234+5.4340\times 10^{-6}\cdot T+1.3154\times 10^{-7}\cdot T^{2}$$

(A17) $$\lambda_{H_{2}O}=0.00053+4.7093\times 10^{-5}\cdot T+4.9551\times 10^{-8}\cdot T^{2}$$

(A18) $$\lambda_{N_{2}}=0.00309+7.5930\times 10^{-5}\cdot T-1.1014\times 10^{-8}\cdot T^{2}$$

The viscosity of mixture gas is derived from References [77,78]:

(A19) $$\lambda_{g}=\sum_{k=1}^{n}\frac{x_{k}\lambda_{k}}{\sum_{j=1}^{n}x_{j}A_{k,j}}$$

(A20) $$A_{k,j}=0.25{\left\{1+{\left[\frac{\mu_{k}}{\mu_{j}}{\left(\frac{M_{j}}{M_{k}}\right)}^{3/4}\frac{T+{\vec{S}}_{k}}{T+{\vec{S}}_{j}}\right]}^{1/2}\right\}}^{2}\frac{T+{\vec{S}}_{k,j}}{T+{\vec{S}}_{j}}$$

where ${\vec{S}}_{k}=1.5\cdot T_{b,k}$, ${\vec{S}}_{k,k}={\vec{S}}_{k}$, ${\vec{S}}_{k,j}={\vec{S}}_{j,k}$, ${\vec{S}}_{k,j}=0.735\sqrt{{\vec{S}}_{k}\cdot {\vec{S}}_{j}}$, $T_{b,k}$ is the boiling temperature of gas except for hydrogen under standard atmosphere pressure, and $T_{{b,H}_{2}}=79\;K$.

Based on the method of Fuller, the molecular diffusion coefficients $D_{k,j}$s of gas pair k−j are estimated as follows [69,70,71]

(A21) $$D_{k,j}=\frac{10^{-7}T^{1.75}{\left(1/M_{k}+1/M_{j}\right)}^{0.5}}{P{\left[{\left(\sum v\right)}_{k}^{1/3}+{\left(\sum v\right)}_{j}^{1/3}\right]}^{2}}\;\;\;\;k,j=1,2,\ldots ,5$$

where ∑v is the diffusion volume, and the detailed data information can be obtained from [71].

The binary diffusion coefficient in the mixture gas are as follows:

(A22) $$D_{m,k}=\sum_{\begin{array}{l} j\neq k\\ j=1 \end{array}}^{5}\frac{D_{k,j}}{x_{j}}\;\;\;\;k,j=1,2,\ldots ,5$$

where $x_{j}$ is the mole fraction of component j.

The pore diffusion due to the collisions between gas molecular and the catalyst pore wall, i.e., the Knudsen diffusion coefficients are estimated as follows [56,77]

(A23) $$D_{N,k}=97\frac{\bar{d_{\mathrm{po}}}}{2}\sqrt{T/M_{k}}\;\;\;\;k=1,2,\ldots ,5$$

where $\bar{d_{\mathrm{po}}}=5\times 10^{-6}$ is the mean pore diameter of the catalyst pellet.

The diffusion coefficient in porous network, i.e., the effective diffusivities $D_{m,k}^{e}$s are depend on the molecular diffusion coefficients $D_{m,k}$s and the Knudsen diffusion coefficients $D_{N,k}$s in single pore:

(A24) $$D_{m,k}^{e}=\frac{\varepsilon_{p}}{\tau }\left(\frac{1}{1/D_{m,k}+1/D_{N,k}}\right)\;\;\;\;k=1,2,\ldots ,5$$

where $\varepsilon_{p}$ and τ are the porosity and tortuosity of catalyst, respectively.
